# Job satisfaction among health professionals in a District of North West province, South Africa

**DOI:** 10.4102/hsag.v28i0.2234

**Published:** 2023-05-22

**Authors:** Reabetswe A. Mere, Thembi V. Simbeni, Mmampedi Mathibe, Mabina N. Mogale, Sam T. Ntuli

**Affiliations:** 1 Department of Public Health, School of Healthcare Sciences, Sefako Makgatho Health Sciences University, Pretoria, South Africa; 2 Department of Statistical Sciences, Faculty of Science and Technology, Sefako Makgatho Health Sciences University, Pretoria, South Africa

**Keywords:** job satisfaction, healthcare professionals, cross-sectional studies, North West Province, working conditions, clinical staff, workforce, motivation

## Abstract

**Background:**

Job satisfaction has become an area of relevance and debate in public health as it is directly linked to staff absenteeism, retention and turnover of the workforce and as such, influences the organisational commitment of the workers and the quality of health services provided. It is therefore essential to discern what drives healthcare professionals to remain working in the public health sector.

**Aim:**

This study aimed to determine job satisfaction and its associated factors among healthcare professionals.

**Setting:**

North-West province South Africa.

**Methods:**

A cross-sectional study was conducted among 244 healthcare professionals of different categories in three district hospitals. A self-administered structured questionnaire with 38 questions to measure job satisfaction was used to collect data. The chi-square test was used to compare groups, and a *p*-value < 0.05 was considered statistically significant.

**Results:**

Overall, 62% of the participants were not satisfied with their job. The most common factors that participants were not satisfied with include job security (52%), standard of care (57%), opportunity to develop (59%), payment or wages (76%), workload (78%) and working environment (89%). Job satisfaction was significantly influenced by age, job category and years of service.

**Conclusions:**

The predictors of job satisfaction include age, category of employees and years of service. Interventions are required to improve the degree of job satisfaction among health care professionals.

**Contribution:**

Findings of this study will assist informing plans that are geared towards enhancing healthcare worker job satisfaction, retention and consequent health systems strengthening.

## Introduction

Healthcare professionals (HPs) have a significant influence on the provision of quality healthcare as well as healthcare access and are thus an important component of the healthcare system. For a health system to function effectively there must be health professionals who are sufficiently skilled, motivated and supported. HPs are the primary drivers of an efficient, effective and sustainable healthcare system (Khunou & Davhana-Maselesele 2016). It is thus vital to comprehend what motivates them to remain working in the public health sector. According to Blaauw et al. (2013), job satisfaction significantly plays a critical role in determining the motivation of the workforce and affects the organisational commitment of healthcare professionals and, as such, the quality of health services provided.

Job satisfaction of healthcare professionals has increasingly become an essential aspect of measure within the South African health system. A comparative study on job satisfaction and intention to leave of different categories of health workers in three Southern African countries indicated that job dissatisfaction was substantially higher in 47.9% of South Africa (SA) than in 29.0% of Malawi and 17.4% of Tanzania (Blaauw et al. 2013). The authors in this study also found that health professionals in SA were more than twice inclined to report an intention to resign as compared to those from the other two countries. In other studies conducted in sub-Saharan Africa, the proportion of healthcare professionals not satisfied with their job ranged from 25.7% to 68.3% (Akuffo et al. 2021; Geta et al. 2021; Merga & Fufa 2019; Mulugeta & Ayele 2015; Temesgen, Aycheh & Leshargie 2018). A similar study conducted among Australian healthcare professionals found that 17.0% of them where not satisfied with their jobs (Scanlan, Devine & Watkins 2021). In India, however, 2.8% of healthcare professionals were not satisfied with their job (Singh et al. 2019).

The World Health Organization’s (WHO) Global strategy on human resources in the health workforce outlined a policy agenda to reach the sustainable development goals (SDGs) targets by 2030 and recommended the optimisation of performance, quality and impact of the health workforce (WHO 2016). Several studies indicated that job satisfaction benefits the health system as it is associated with building up employee motivation, performance, efficiency and retention (Blaauw et al. 2013; Merga & Fufa 2019). Furthermore, it assists organisations in achieving their objectives and goals. According to Mbindyo et al. (2009), the motivation of healthcare workers could lay the foundation for pushing and maintaining efforts towards reaching SDGs. Evaluation of job satisfaction in the healthcare sector is not a new phenomenon worldwide. In SA, studies have been conducted to evaluate job satisfaction and its associated factors among healthcare professionals; however, most of these studies concentrated mainly on professional nurses (Khamisa et al. 2015; Khunou & Davhana-Maselesele 2016; Morton et al. 2020; Payne et al. 2020). There is a paucity of information on the level of job satisfaction in all categories of healthcare professionals. Thus, this study’s objectives were to determine job satisfaction and to determine factors associated with job satisfaction among all categories of healthcare professionals in Ngaka Modiri District in North-West province.

## Research methods and design

### Study design and setting

This cross-sectional study was conducted between December and January 2021 at Ngaka Modiri Molema District of the North West province SA. The study was conducted in three randomly selected public hospitals from four hospitals in the region. The total number of healthcare professionals from the three institutions was 393, with 196 at Zeerust/Lehurutshe Complex Hospital, 126 at Thusong/General De La Rey Complex Hospital and 71 at Gelukspan District Hospital.

### Study population

The study population comprised all categories of healthcare professionals (i.e. medical practitioners, professional nurses, dieticians, physiotherapists, pharmacists, occupational therapists, radiographers and clinical associates) who were on duty during the data-collection period.

### Inclusion and exclusion criteria

Healthcare professionals who served for less than 12 months in the hospital at the time of data collection, work part-time and were unwilling to give informed consent were excluded from the study. All the above-mentioned categories of health professionals, who have been on full-time employment for 12 months and more and willing to participate were included in the study.

### Sampling and sample size

The required sample size for this study was determined using Yamane’s (1967) formula:


$$n=\frac{N}{1+N{\left(e\right)}^{2}}=198$$ [Eqn 1]

Where:

*n* is the sample size

*N* is the population size of the three hospitals (393)

*e* is the sampling error (5%).

A non-response rate of 10% was expected, and the sample size was increased to 218. The facilities have different numbers of healthcare professionals. Thus, the sample was allocated proportionally to the hospitals. To distribute the tools to all categories of healthcare professionals, participants were grouped according to their professional category, and then from each group, a consecutive sampling technique was used to administer the questionnaire.

### Recruitment

The researchers were responsible for recruiting study participants and coordinating the consenting process. The researchers recruited healthcare professionals during their tea times at their respective working stations by giving verbal information about the study and its purpose. Recruitment was done during the day and at night, in order to include all healthcare professionals who meet the criteria and are willing to participate in the study.

### Data collection

A structured self-administered questionnaire was used for data collection, which is developed using relevant literature (Khamisa et al. 2015; Khunou & Davhana-Maselesele 2016; Morton et al. 2020; Payne et al. 2020). The tool includes demographic profiles such as gender, age, marital status, professional category, level of education and years of experience as a healthcare professional.

The second part of the questionnaire had 38 questions used to measure job satisfaction and answered as either dissatisfied 0, unsure 0 or satisfied 1 (dissatisfied was merged with unsure). The section that measures job satisfaction has nine dimensions: personal satisfaction (six items), workload (four items), staff relations (four items), opportunity to develop (five items), payment or wages (three items), job security (three items), standards of care (four items), leadership and supervision (five items) and working environment (four items). Data collection occurred for a period of four weeks from the first week of December to 29 December in year 2020.

### Data analysis

Data were entered and analysed using the Microsoft Excel© Spreadsheet and statistical package STATA version 16, respectively. The total score for each item in the dimension of satisfaction was dichotomised into satisfied and dissatisfied using the mean score as a cut-off point, so a score equal to or above the mean value was considered satisfied otherwise dissatisfied. Comparison between the two groups was performed using the chi-square test. A *p*-value of less than 0.05 was considered statistically significant.

### Reliability and validity

The reliability coefficient values ranged from 0.736 – 0.964, which are considered to range from acceptable to excellent (Table 1).

**TABLE 1:** Cronbach’s alpha coefficient.

Job satisfaction components	Number of items	Cronbach’s alpha
Personal satisfaction	6	0.878
Workload	4	0.848
Staff relations	4	0.758
Opportunity to develop	5	0.869
Payment or wages	3	0.860
Job security	3	0.840
Standards of care	4	0.900
Leadership and supervision	5	0.878
Working environment	4	0.736

### Ethical considerations

The Research Ethics Committee of Sefako Makgatho Health Sciences University gave ethical approval (SMUREC/H/172/2020) as this study was part of a Master of Public Health (MPH) research project. Permission to carry out this study was sought from the North West Department of Health and the managers of the three district hospitals where the study was being conducted. All the participants completed the informed consent and were assured that the data collected would be kept confidential and used only for this study. Participants were also informed that participation was voluntary and they were free to withdraw from the study at any time. Consent to publish study findings was also obtained from the participants.

## Results

### Demographic characteristics

A total of 244 healthcare professionals participated in this study. Their mean age was 40.3 ± 11.1 years ranging from 23 – 64 years. More than two-thirds (75%) of the participants were aged less than 50 years and 66% were females. Slightly more than half (54%) were married and 75% were clinical staff (Table 2). The mean number of years working as a health care professional was 11.6 ± 8.4 years ranging from 1 – 36 years. Nearly half (45%) of the participants had more than 10 years of working experience as healthcare professionals.

**TABLE 2:** Demographic characteristics of the participants, *n* = 244.

Socio-demographic variables	Frequency	Percentage (%)
**Age (years)**		
< 30	49	20
30–39	72	30
40–49	61	25
≥ 50	63	22
**Gender**		
Male	83	34
Female	161	66
**Marital status**		
Single	111	46
Married	133	54
**Job category**		
Clinical staff	184	75
Clinical support staff	60	25
**Years of experience**		
< 5	56	23
5–10	78	32
> 10	110	45

### Level of job satisfaction among health professionals

**FIGURE 1:**
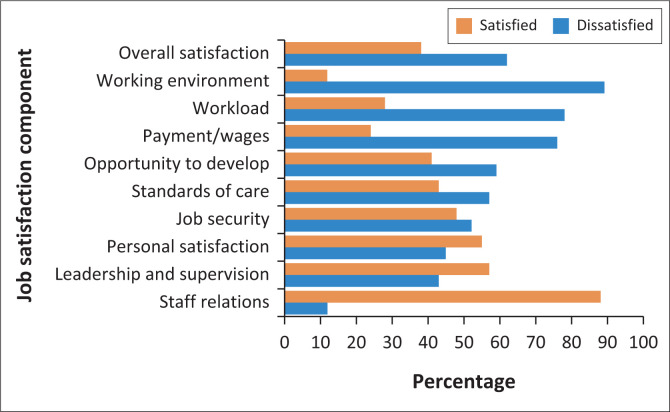
Satisfaction level of health professionals with different components of the hospital.

Overall, 62% of the participants were found to be dissatisfied with their job. The most common aspects that the participants were not satisfied with include: 52% job security, 57% standard of care, 59% opportunity to develop, 76% payment or wages, 78% workload and 89% working environment (Figure 1).

To determine items affecting job satisfaction under each dimension, the results showed that for the work environment dimension with four items, 91% availability of equipment, 87% state of hospital infrastructure and 84% maintenance of the hospital were the main contributors to job dissatisfaction. Meanwhile, for the workload dimension, all four items were identified to contribute to job dissatisfaction, 79% overall staffing levels, 76% my workload, 64% time available to get through my work and 62% of the hours I work.

In terms of payments or wages dimension with three items, 77% degree to which I am fairly paid and 75% of my salary or pay scale were found to be the main causes of job dissatisfaction. Five items for the opportunity to develop dimension are ordered from the highest to the lowest causes of job dissatisfaction: being funded for courses, opportunities I’ve to advance my career or prospects for promotion, opportunity to attend courses, time off for in-service training and extent to which I’ve adequate training for what I do.

### Factors associated with overall job satisfaction among healthcare professionals

As shown in Table 3, there is no statistically significant association between gender (*p* > 0.05) and marital status (*p* > 0.05) with the overall level of satisfaction. A significantly greater proportion of healthcare professionals under 30 years were likely to be dissatisfied with their job as compared to the other age groups (*p* < 0.05). The result also showed that clinical staff and those with less than 5 years of working experience as healthcare professionals were significantly likely to be dissatisfied with their job (*p* < 0.05).

**TABLE 3:** Association between demographics and overall level of satisfaction.

Socio-demographic variables	Level of job satisfaction		*p*
	Dissatisfied	Satisfied	
**Age**			
< 30	39 (80)	10 (20)	0.026
30–39	42 (58)	30 (42)	
40–49	32 (53)	29 (47)	
50+	37 (60)	25 (40)	
**Gender**			
Male	46 (55)	37 (45)	0.163
Female	104 (65)	57 (35)	
**Marital status**			
Single	63 (57)	48 (43)	0.166
Married	87 (65)	46 (35)	
**Job category**			
Clinical staff	122 (66)	62 (34)	0.007
Clinical support staff	28 (47)	32 (53)	
**Years of experience**			
< 5	43 (77)	13 (23)	0.020
5–10	47 (60)	31 (40)	
> 10	60 (55)	50 (45)	

Table 4 shows the association between demographics and job satisfaction dimensions. The result revealed that the personal satisfaction dimension is statistically associated with age (*p* = 0.04) and years of working experience as a healthcare professional (*p* = 0.029). Participants aged less than 30 years and those with less than 5 years of working experience as healthcare professionals were more likely to be dissatisfied than the other groups.

**TABLE 4:** Association of the demographic characteristics and job satisfaction dimensions.

Socio-demographic variables	*n*	Personal satisfaction	*p*-value	Workload	*p*-value	Staff relations	*p*-value	Opportunity to develop	*p*-value	Payments	*p*-value	Job security	*p*-value	Standards of care	*p*-value	leadership and supervision	*p*-value	Working environment	*p*-value
**Age**																			
< 30	49	29 (59)	0.04	35 (71)	0.015	12 (25)	0.032	40 (82)	0.001	38 (78)	0.912	32 (65)	0.002	31 (63)	0.08	23 (57)	0.096	46 (94)	0.416
30–39	72	36 (50)		49 (68)		8 (11)		45 (63)		53 (74)		32 (44)		46 (64)		32 (44)		64 (89)	
40–49	61	23 (38)		54 (89)		5 (8)		31 (51)		48 (79)		22 (36)		35 (57)		21 (34)		51 (84)	
50+	63	22 (36)		52 (84)		5 (8)		29 (47)		47 (76)		40 (65)		27 (44)		24 (39)		55 (89)	
**Gender**																			
Male	83	34 (41)	0.353	61 (74)	0.237	8 (10)	0.364	52 (63)	0.461	57 (69)	0.047	37 (45)	0.113	50 (60)	0.458	39 (47)	0.37	69 (83)	0.058
Female	161	76 (47)		129 (80)		22 (14)		93 (58)		129 (80)		89 (55)		89 (55)		66 (41)		147 (91)	
**Marital Status**																			
Single	111	46 (41)	0.296	87 (78)	0.861	9 (8)	0.069	60 (54)	0.118	76 (69)	0.009	47 (42)	0.008	65 (59)	0.646	48 (43)	0.952	99 (89)	0.766
Married	133	64 (48)		103 (77)		21 (16)		85 (64)		110 (83)		79 (59)		74 (56)		57 (43)		117 (88)	
**Job category**																			
Clinical staff	184	87 (47)	0.226	174 (95)	<0.001	24 (13)	0.533	99 (54)	0.007	154 (84)	<0.001	101 (55)	0.075	101 (55)	0.251	84 (46)	0.148	160 (87)	0.178
Clinical support staff	60	23 (38)		16 (27)		6 (10)		46 (77)		32 (53)		25 (42)		38 (63)		21 (35)		56 (93)	
**Years of experience**																			
< 5	56	33 (59)	0.029	40 (71)	0.073	13 (23)	0.004	46 (82)	<0.001	39 (70)	0.328	36 (64)	0.086	34 (61)	0.128	31 (55)	0.098	53 (95)	0.258
5–10	78	36 (46)		57 (73)		11 (14)		50 (64)		63 (81)		39 (50)		50 (64)		32 (41)		68 (87)	
> 10	110	41 (37)		93 (85)		6 (6)		49 (45)		84 (76)		51 (46)		55 (50)		42 (38)		95 (86)	

The clinical staff (*p* < 0.001) and those aged 40 years and older (*p* = 0.015) were significantly dissatisfied with the dimension of the workload. The results also showed that participants aged < 30 years (*p* = 0.032) and those with less than 5 years of working experience as healthcare professionals (*p* = 0.004) were more likely to be dissatisfied with the staff relation dimension. The dimension of opportunity to develop was found to be statistically related to age (*p* = 0.001), job category (*p* = 0.007) and years of experience (*p* < 0.001). The younger age group, clinical support staff and those with less working experience were dissatisfied with the dimension of opportunity to develop.

Concerning the payments or wages dimension, female gender (*p* = 0.047), married (*p* = 0.009) and clinical staff (*p* < 0.001) were significantly dissatisfied with the dimension of payments or wages. The result revealed no statistically significant relationship between age (*p* = 0.08), gender (*p* = 0.458), marital status (0.646), job category (*p* = 0.251), years of experience (*p* = 0.128) and standard of care dimension. The leadership and supervision dimension were also found not significantly associated with age (*p* = 0.096), gender (*p* = 0.370), marital status (0.952), job category (*p* = 0.148) and years of experience (*p* = 0.098). Similarly, the finding also indicates no statistically significant association between age (*p* = 0.416), gender (*p* = 0.058), marital status (0.766), job category (*p* = 0.178), years of experience (*p* = 0.258) and the dimension of a working environment.

## Discussion

The purpose of this study was to determine the level of job satisfaction and its associated factors among healthcare professionals in the Ngaka Modiri Molema District, North West province The finding revealed that nearly two-thirds (62%) of the participants were found not to be satisfied with their job. This finding is similar to studies conducted in Ethiopia that reported that 61.5% (Merga & Fufa 2019) and 65.1% (Mulugeta & Ayele 2015) of healthcare professionals were not satisfied with their job. The rate of job dissatisfaction observed in our study is higher than 44.8% (Geta et al. 2021), 25.7% (Akuffo et al. 2021), 17.0% (Scanlan et al. 2021), 14.8% (Qiu et al. 2021) and 2.8% (Singh et al. 2019) reported in previous similar studies. However, the finding of this study is slightly lower than the 68.3% found in a cross-sectional study conducted among healthcare professionals working in public hospitals in Ethiopia (Temesgen et al. 2018). The high job dissatisfaction rate observed in the present study is a cause for concern as dissatisfied healthcare professionals provide substandard and less effective healthcare.

In the present study, the main source of job dissatisfaction identified was the work environment, which accounted for more than two-thirds (89%) of the dissatisfied healthcare professionals. Our finding is similar to the findings of previous studies that reported that poor job satisfaction is because of the work environment (Akuffo et al. 2021; Bonenberger et al. 2014; Kumar et al. 2013; Mulugeta & Ayele 2015). Employees usually attain a feeling of satisfaction with their jobs when they are working in a clean environment with all adequate commodities and supplies as well as acceptable levels of environment and temperature (Munyewende, Rispel & Chirwa 2014).

Dissatisfaction with the workload is another important issue affecting 78% of healthcare professionals in our study. A similar finding was reported among nurses working in tertiary care hospitals in India (Gulavani & Shinde 2014) and medical practitioners and nurses in China (Jin et al. 2019). A high workload is associated with inappropriate treatment (Guan et al. 2021; Yang et al. 2019) and can be minimised by addressing the shortage of staff, proper planning of duty schedules and training staff to plan their priorities.

Consistent with a number of studies, most (76%) of the healthcare professionals were not satisfied with their wage studies (Akuffo et al. 2021; Anand et al. 2022; Asegid et al. 2014; Ayalew et al. 2019; Khunou & Davhana-Maselesele 2016; Kumar et al. 2013;). Khunou and Davhana-Maselesele (2016) stated that since the implementation of Occupational Specific Dispensation (OSD), no studies were conducted regarding the impact of this policy on the level of job satisfaction among healthcare professionals. Other essential factors that influence job satisfaction in the present study were job security, the standard of care and lack of opportunity to develop. Mengistu and Bali (2015) in their study found that lack of training opportunities, poor performance evaluation systems and poor working conditions were the main cause of job health professionals not being satisfied with their job.

Various socio-demographic characteristics such as age, sex, level of education and years of experience as healthcare professionals were found to be associated with job satisfaction (Asegid et al. 2014; Ayalew et al. 2019; Lu et al. 2016). However, other studies reported no association between socio-demographic factors and job satisfaction (Chaulagain & Khadkas 2012; Elsherbeny & El-Masry 2018). In this study, job satisfaction significantly increases with age and years of experience and is higher among clinical support staff. Concerning the relationship between demographics and job satisfaction dimensions, the study revealed that participants aged < 30 years were significantly not satisfied with personal satisfaction, workload, opportunity to develop and job security. In addition, female gender and married participants were significantly not satisfied with payment or wages, while the married respondents were also not happy with job security. Clinical staff were significantly unhappy with the workload, while clinical support staff were not satisfied with the opportunity to develop.

### Study limitations

This study was conducted in three hospitals in the province which makes it difficult to generalise the findings of this study to the entire province. Another limitation is that the studies reviewed used different questionnaires to assess job satisfaction; moreover, the definitions of job satisfaction are different.

## Conclusion

In conclusion, the findings of this study indicated that the healthcare professionals in these settings had a low level of job satisfaction. Working environment, workload, payment or wages, development opportunities, standards of care and job security were the most common dimensions that cause participants to remain not satisfied. In addition, demographic factors such as age, job category and years of service influence job satisfaction. The policymakers, healthcare managers and other stakeholders must establish and implement effective policies that will increase the job security of healthcare professionals and decrease their intention to leave their positions by enhancing their working conditions and job satisfaction, paying them fairly, fostering job prospects and stability and supporting competency-based career advancement. Additionally, the workload should be reduced by effective duty schedule planning, a decrease in healthcare professional turnover, greater recruitment of trained and experienced personnel and training of employees in the technique of prioritisation of activities.
